# Supplementary material for: Granulosa cell transcription is similarly impacted by superovulation and aging and predicts early embryonic trajectories
Source: Nat Commun. 2025 Apr 17;16:3658. doi: 10.1038/s41467-025-58451-9 (PMC12006393; doi:10.1038/s41467-025-58451-9)
Supplement: Supplementary file 1 — Supplementary Information [file 41467_2025_58451_MOESM1_ESM.pdf]

Granulosa cell transcription is similarly impacted by superovulation and aging and predicts early embryonic trajectories

Klaudija Daugelaite[1,2,#], Perrine Lacour[2,3,#], Ivana Winkler[3,#], Marie-Luise Koch[1], Anja Schneider[1], Nina Schneider[3], Francesca Coraggio[3], Alexander Tolkachov[1,4], Xuan Phuoc Nguyen[5], Adriana Vilkaite[5], Julia Rehnitz[5], Duncan T. Odom[1,*], Angela Goncalves[3,*]

# These authors contributed equally: Klaudija Daugelaite, Perrine Lacour, Ivana Winkler

* Corresponding authors: Duncan T. Odom (d.odom@dkfz.de), Angela Goncalves (a.goncalves@dkfz.de)

Affiliations

1. German Cancer Research Center (DKFZ), Division of Regulatory Genomics and Cancer Evolution, Heidelberg, Germany

2. Faculty of Biosciences, Ruprecht-Karl-University Heidelberg, Heidelberg, Germany

3. German Cancer Research Center (DKFZ), Division of Somatic Evolution and Early Detection, Heidelberg, Germany

4. Current address: Department of Cardiology, Angiology and Pneumology, Internal Medicine III, Heidelberg University Hospital, Heidelberg, Germany

5. Department of Gynecological Endocrinology and Fertility Disorders, University Women's Hospital Heidelberg, Heidelberg, Germany

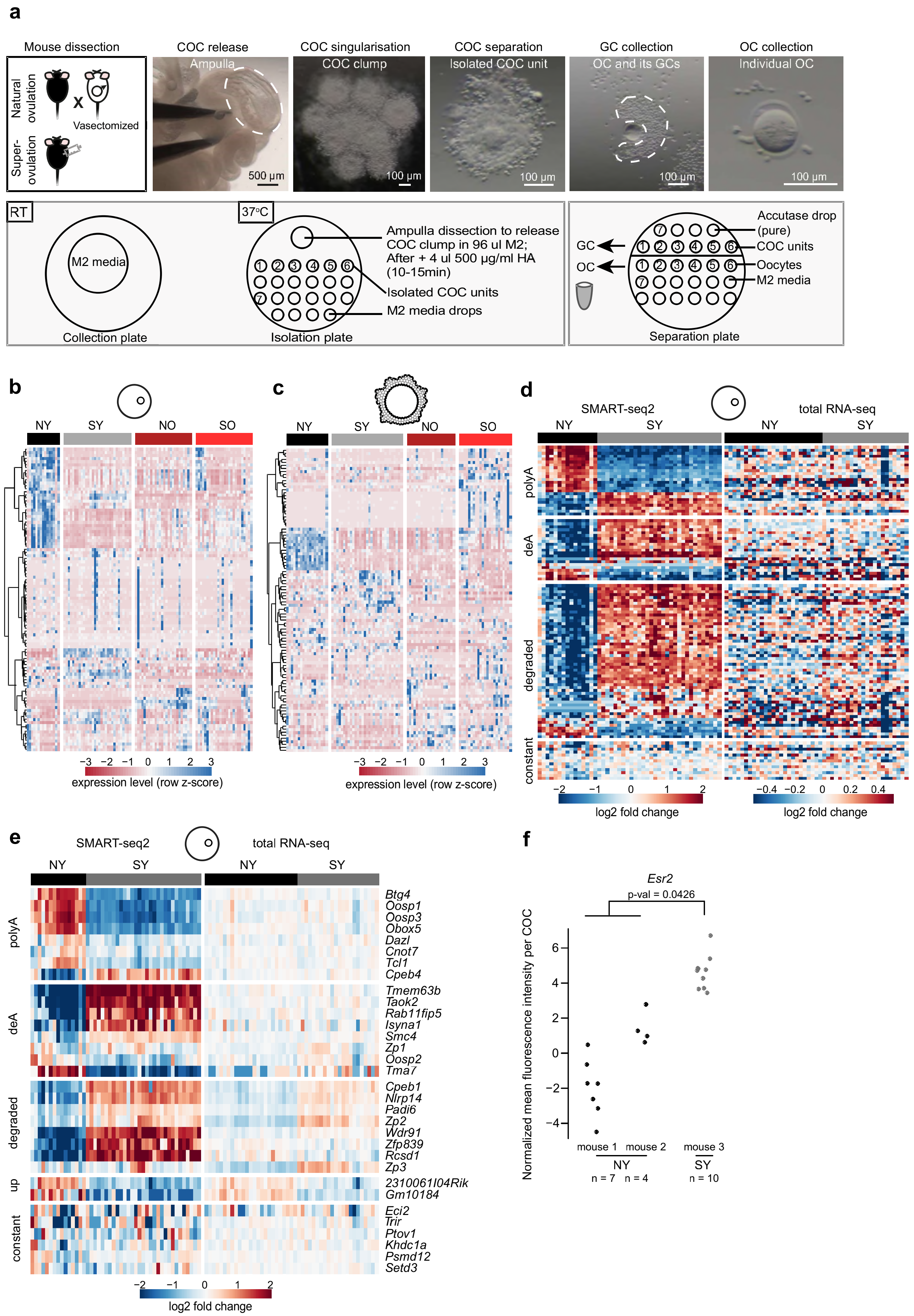
a
Mouse dissection
Natural ovulation
Vasectomized
Super-ovulation
COC release
Ampulla
500 µm
COC singularisation
COC clump
100 µm
COC separation
Isolated COC unit
100 µm
GC collection
OC and its GCs
100 µm
OC collection
Individual OC
100 µm
RT
M2 media
Collection plate
37°C
Ampulla dissection to release COC clump in 96 ul M2; After + 4 ul 500 µg/ml HA (10-15min)
Isolated COC units
M2 media drops
Isolation plate
GC
OC
Accutase drop (pure)
COC units
Oocytes
M2 media
Separation plate
b
NY SY NO SO
expression level (row z-score)
c
NY SY NO SO
expression level (row z-score)
d
SMART-seq2
total RNA-seq
NY SY NY SY
polyA
deA
degraded
constant
log2 fold change
log2 fold change
e
SMART-seq2
total RNA-seq
NY SY NY SY
polyA
deA
degraded
up
constant
Btg4
Oosp1
Oosp3
Obox5
Dazl
Cnot7
Tcl1
Cpeb4
Tmem63b
Taok2
Rab11fip5
Isyna1
Smc4
Zp1
Oosp2
Tma7
Cpeb1
Nlrp14
Padi6
Zp2
Wdr91
Zfp839
Rcsd1
Zp3
2310061I04Rik
Gm10184
Eci2
Trir
Ptov1
Khdc1a
Psmd12
Setd3
log2 fold change
f
Esr2
p-val = 0.0426
Normalized mean fluorescence intensity per COC
mouse 1 mouse 2 mouse 3
NY SY
n = 7 n = 4 n = 10

Supplementary Figure 1. Collection of cumulus-oocyte-complexes from young and old mice after natural and superovulation reveals that key maturation pathways are dysregulated after superovulation

(a) Experimental protocol for cumulus-oocyte complex (COC) singularization and paired oocyte-granulosa (OC-GC) cell separation. Experiments were repeated for each individual mouse (n = 37), representing a total of approximately 551 times. **(b,c)** Top 100 most variable genes in oocytes (b) and granulosa cells (c) in naturally ovulated young (NY, black, n = 15 OC, 18 GC), superovulated young (SY, grey, n = 31 OC, 31 GC), naturally ovulated old (NO, dark red, n = 26 OC, 21 GC), superovulated old (SO, light red, n = 26 OC, 23 GC). **(d)** Comparison of gene expression between naturally and superovulated oocytes using SMART-seq2 (n = 15 NY, 31 SY oocytes) and total RNA-seq (n = 25 NY, 22 SY oocytes) for genes from Lee et al., 2024[1] (Methods, polyA: re-poly-adenylated, deA: de-adenylated). For each technology, the fold change is computed between the two groups. Each column represents gene expression in an individual oocyte. **(e)** Comparison of gene expression between naturally and superovulated oocytes using SMART-seq2 (n = 15 NY, 31 SY oocytes) and total RNA-seq (n = 25 NY, 22 SY oocytes) for extended list of genes shown in Figure 2d. For each technology, the fold change is computed between the two groups (Methods). Each column represents gene expression in an individual oocyte. **(f)** *Esr2* gene expression quantified from fluorescence intensity in granulosa cells from young naturally and superovulated mice (n = 2 NY, 1 SY mice, number of COC indicated in the figure). Normalization is done on a sample without *Esr2* probes (Methods). P-value is estimated by comparing two linear models, with and without treatment effect (Methods).

Source data are provided as a Source Data file.

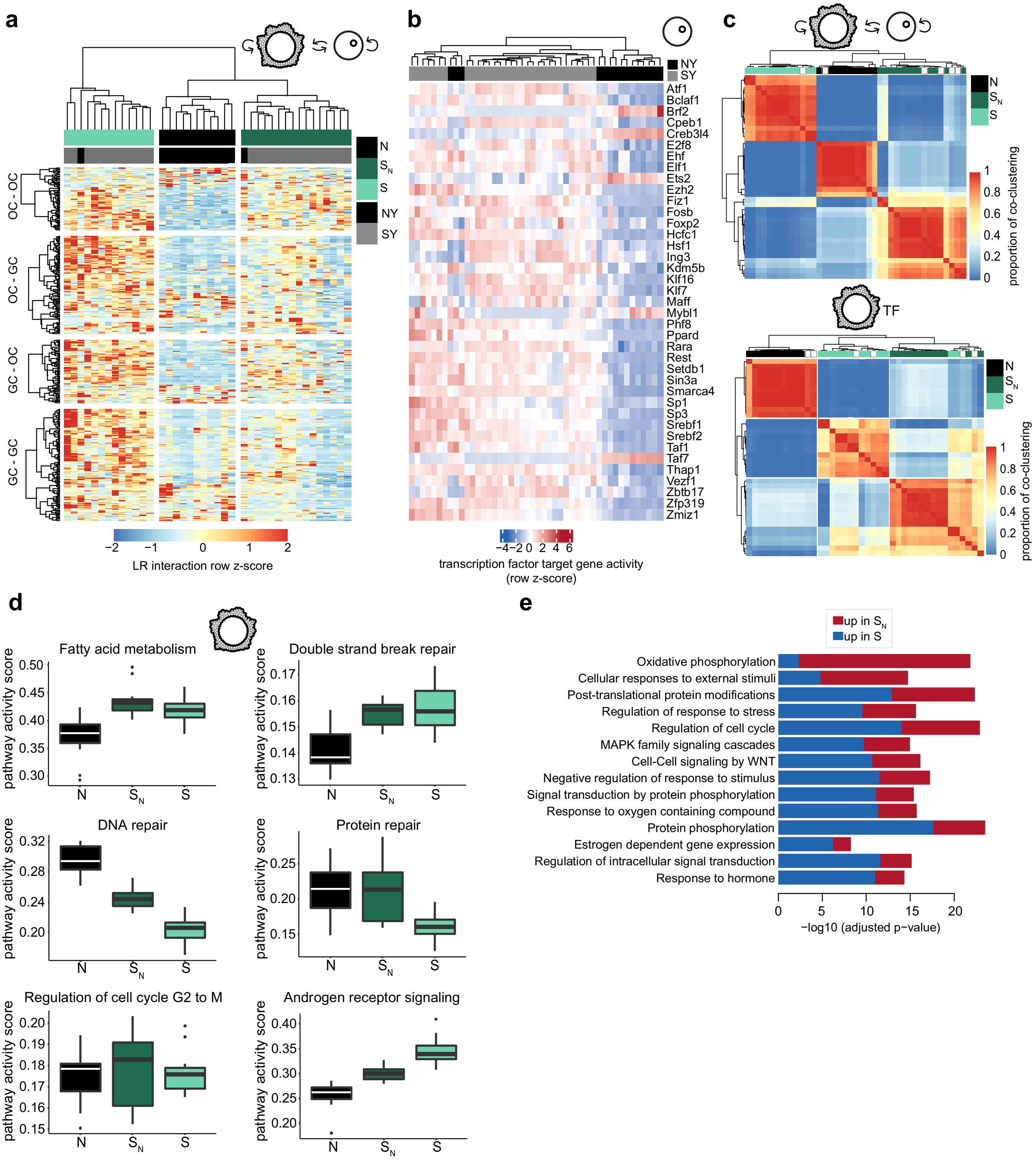
a
N
S N
S
NY
SY
OC - OC
OC - GC
GC - OC
GC - GC
−2 −1 0 1 2
LR interaction row z-score
b
NY
SY
Atf1
Bclaf1
Brf2
Cpeb1
Creb3l4
E2f8
Ehf
Elf1
Ets2
Ezh2
Fiz1
Fosb
Foxp2
Hcfc1
Hsf1
Ing3
Kdm5b
Klf16
Klf7
Maff
Mybl1
Phf8
Ppard
Rara
Rest
Setdb1
Sin3a
Smarca4
Sp1
Sp3
Srebf1
Srebf2
Taf1
Taf7
Thap1
Vezf1
Zbtb17
Zfp319
Zmiz1
−4 −2 0 2 4 6
transcription factor target gene activity
(row z-score)
c
N
S N
S
1
0.8
0.6
0.4
0.2
0
proportion of co-clustering
TF
d
Fatty acid metabolism
Double strand break repair
DNA repair
Protein repair
Regulation of cell cycle G2 to M
Androgen receptor signaling
pathway activity score
N
S N
S
e
up in S N
up in S
Oxidative phosphorylation
Cellular responses to external stimuli
Post-translational protein modifications
Regulation of response to stress
Regulation of cell cycle
MAPK family signaling cascades
Cell-Cell signaling by WNT
Negative regulation of response to stimulus
Signal transduction by protein phosphorylation
Response to oxygen containing compound
Protein phosphorylation
Estrogen dependent gene expression
Regulation of intracellular signal transduction
Response to hormone
0 5 10 15 20
−log10 (adjusted p−value)

Supplementary Figure 2. Superovulation dysregulates cell-to-cell communication and critical pathways' activities in granulosa cells

(a) Cell-cell communication scores for all ligand-receptor interactions tested. Each row represents a ligand-receptor interaction and each column is a granulosa-oocyte pair (n = 13 NY, black, 27 SY, grey, OC-GC pairs). Clusters are marked as N (black), S_N (dark green), S (light green). **(b)** Activity scores of transcription factors associated with enriched pathways shown in Figure 2a in oocytes. TFs whose activity scores were significantly different between naturally ovulated (NY, black, n = 15 oocytes) and superovulated young (SY, grey, n = 31 oocytes) oocytes by a two-tailed permutation test are shown (adjusted p-value < 0.05). **(c)** Stability of the clusters obtained from cell-cell communication (top) and transcription factor (bottom) analyses assessed using bootstrapping. The heatmap color represents how often the two samples cluster together. **(d)** Activity scores of enriched pathways from Figure 2b in granulosa cells split by consensus clusters N, S_N and S identified in Figures 3a and 3d (n = 10 N, 12 S_N, 12 S). **(e)** Overrepresentation analysis of genes differentially expressed between S and S_N granulosa cells. The bars are colored based on the proportion of genes that are up- or down-regulated. P-values were estimated using a one-tailed hypergeometric test and corrected for multiple testing.

Source data are provided as a Source Data file.

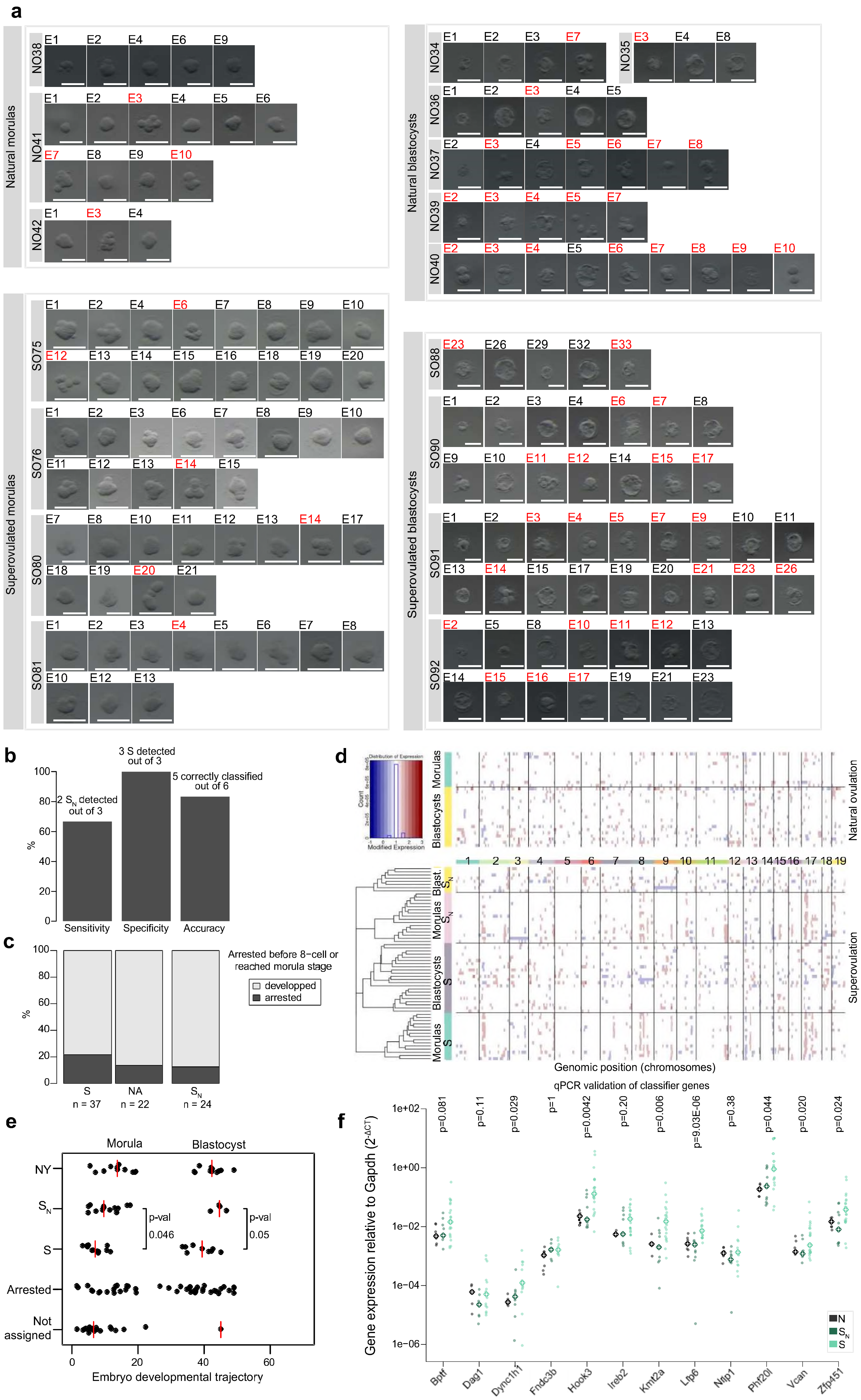
a
Natural morulas
NO38
NO41
NO42
Natural blastocysts
NO34
NO35
NO36
NO37
NO39
NO40
Superovulated morulas
SO75
SO76
SO80
SO81
Superovulated blastocysts
SO88
SO90
SO91
SO92
b
3 S detected out of 3
5 correctly classified out of 6
2 S_N detected out of 3
%
Sensitivity
Specificity
Accuracy
c
Arrested before 8−cell or reached morula stage
developped
arrested
%
S n = 37
NA n = 22
S_N n = 24
d
Distribution of Expression
Count
Modified Expression
Morulas
Blastocysts
Natural ovulation
Blast. S_N
Morulas S_N
Blastocysts S
Morulas S
Superovulation
1 2 3 4 5 6 7 8 9 10 11 12 13 14 15 16 17 18 19
Genomic position (chromosomes)
e
Morula
Blastocyst
NY
S_N
S
Arrested
Not assigned
p-val 0.046
p-val 0.05
Embryo developmental trajectory
f
qPCR validation of classifier genes
Gene expression relative to Gapdh ($2^{-\Delta CT}$)
p=0.081
p=0.11
p=0.029
p=1
p=0.0042
p=0.20
p=0.006
p=9.03E-06
p=0.38
p=0.044
p=0.020
p=0.024
Bptf
Dag1
Dync1h1
Fndc3b
Hook3
Ireb2
Kmt2a
Lrp6
Nfrp1
Phf20l
Vcan
Zfp451
N
S_N
S

Supplementary Figure 3. Classification of granulosa cells to study the pre-implantation development of their associated embryos

(a) Widefield images of all embryos collected at the morula (62-64h post-fertilization) and blastocyst (108h post-fertilization) stages. Embryos which arrested before the target stage are labeled in red. All scale bars represent 100µm. LUTs were not applied, in several images the amount of black and white were reduced to improve visibility. Embryos from each individual mouse are marked by corresponding mouse ID. **(b)** Specificity, sensitivity and accuracy of the granulosa cell classifier on the test set. **(c)** Proportion of embryos that arrested (dark grey) before morula stage in S, S_N, and not assigned (NA) groups compared to the ones that reached the morula stage (light grey). The Fisher test p-value is not significant due to the small number of embryos. **(d)** Whole genome copy number representation from inferCNV in the superovulated embryos (lower panel) using embryos derived from natural ovulation as a reference (upper panel, Methods). A few embryos were identified as aneuploid but no pattern relative to the classification of their associated granulosa cells is observable. **(e)** Embryos classified by their granulosa cell class along pseudotime calculated on developmental genes (n = 22 NY, 17 S_N, 21 S, 19 NA, 44 arrested embryos). Red line - median, two-tailed Wilcoxon test was used to compute p-values. **(f)** Gene expression of tested classifier genes in young natural (NY), S_N and S granulosa cells measured by qPCR (n = 7 NY, 11 S_N and 23 S granulosa cells). Empty circles represent the median, two-tailed Wilcoxon test was performed between S_N and S groups to determine p-values.

Source data are provided as a Source Data file.

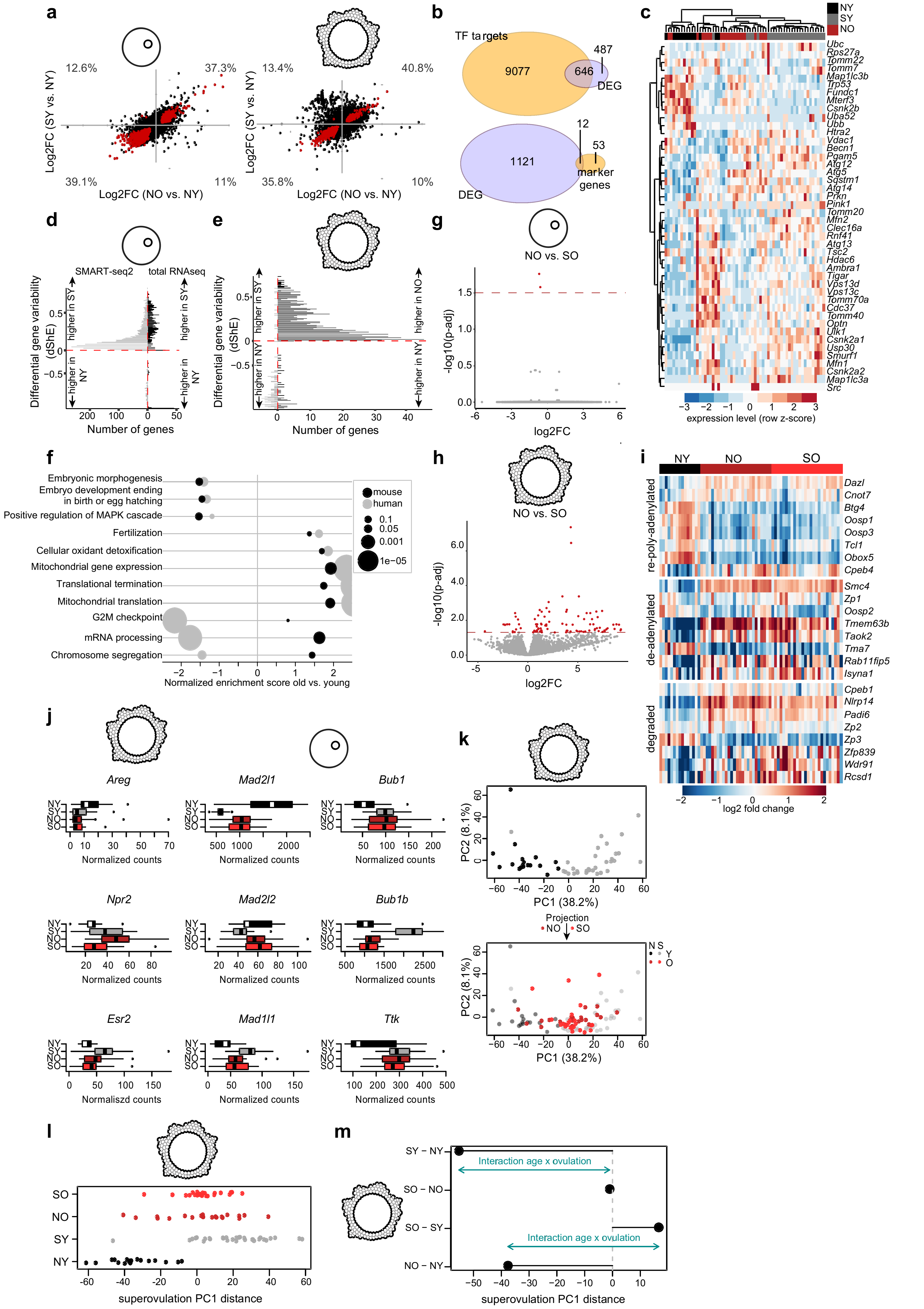

a
12.6%
37.3%
39.1%
11%
Log2FC (SY vs. NY)
Log2FC (NO vs. NY)
13.4%
40.8%
35.8%
10%
b
TF targets
9077
646
487
DEG
1121
12
53
marker genes
c
NY
SY
NO
Ubc
Rps27a
Tomm22
Tomm7
Map1lc3b
Trp53
Fundc1
Mterf3
Csnk2b
Uba52
Ubb
Htra2
Vdac1
Becn1
Pgam5
Atg12
Atg5
Sqstm1
Atg14
Prkn
Pink1
Tomm20
Mfn2
Clec16a
Rnf41
Atg13
Tsc2
Hdac6
Ambra1
Tigar
Vps13d
Vps13c
Tomm70a
Cdc37
Tomm40
Optn
Ulk1
Csnk2a1
Usp30
Smurf1
Mfn1
Csnk2a2
Map1lc3a
Src
expression level (row z-score)
d
SMART-seq2
total RNAseq
higher in SY
higher in NY
Differential gene variability (dShE)
Number of genes
e
higher in NO
g
NO vs. SO
-log10(p-adj)
log2FC
f
Embryonic morphogenesis
Embryo development ending in birth or egg hatching
Positive regulation of MAPK cascade
Fertilization
Cellular oxidant detoxification
Mitochondrial gene expression
Translational termination
Mitochondrial translation
G2M checkpoint
mRNA processing
Chromosome segregation
mouse
human
0.1
0.05
0.001
1e−05
Normalized enrichment score old vs. young
h
NO vs. SO
-log10(p-adj)
log2FC
i
NY
NO
SO
re-poly-adenylated
Dazl
Cnot7
Btg4
Oosp1
Oosp3
Tcl1
Obox5
Cpeb4
de-adenylated
Smc4
Zp1
Oosp2
Tmem63b
Taok2
Tma7
Rab11fip5
Isyna1
degraded
Cpeb1
Nlrp14
Padi6
Zp2
Zp3
Zfp839
Wdr91
Rcsd1
log2 fold change
j
Areg
Mad2l1
Bub1
Npr2
Mad2l2
Bub1b
Esr2
Mad1l1
Ttk
NY
SY
NO
SO
Normalized counts
Normaliszd counts
k
PC2 (8.1%)
PC1 (38.2%)
Projection
NO
SO
l
superovulation PC1 distance
m
SY − NY
SO − NO
SO − SY
NO − NY
Interaction age x ovulation
superovulation PC1 distance

Supplementary Figure 4. Superovulation and aging have similar but non-additive effects on the transcriptomes of oocytes and granulosa cells

(a) Log2 fold change of gene expression in superovulated (SY) versus naturally ovulated young (NY) compared to Log2 fold change in naturally ovulated old (NO) versus young (NY) oocytes and granulosa cells (n = 3 NY, 4 SY, 3 NO mice). Red dots are the genes from Figures 5b and 5c. Percentages represent the proportion of genes in each quadrant. **(b)** Overlap between the genes used in the two classifiers (orange) in Figure 4a ('TF targets' - first classifier based on SCENIC regulons, 'marker genes' - second classifier based on DESeq2 genes, see Methods) with the genes deregulated by both aging and superovulation from Figure 5c ('DEG', violet). **(c)** Expression of genes involved in mitophagy pathways in NY, SY and NO oocytes. The heatmap is clustered by rows and columns. **(d)** Comparison of differential Shannon's entropy (dShE) results in oocytes between two different sequencing methods - SMART-seq2 (left, n = 15 NY and 31 SY oocytes) and total RNA-seq (right, n = 25 NY and 22 SY oocytes). **(e)** Distribution of differential gene variability (dShE) in SY (left) and NO (right) in comparison to NY granulosa cells (n = 18 NY, 31 SY, 21 NO granulosa cells). **(f)** Overrepresentation analysis of pathways in aging mouse oocytes (our dataset) and human oocytes (Ntostis et al., 2022[2]). The size of the circle represents adjusted p-values estimated using an adaptive multi-level split Monte-Carlo scheme (implemented in the fgsea package). **(g, h)** Differential expression analysis of superovulated old (SO) versus naturally ovulated old (NO) oocytes (g) and granulosa cells (h) reveals few significant genes (Wald test implemented in DESeq2, adjusted p-value < 0.05, n = 3 NO, 3 SO mice). **(i)** Comparison of gene expression between NY, NO and SO oocytes using SMART-seq2. The fold change is computed between the old and young groups. Each column represents gene expression in an individual oocyte. **(j)** Gene expression of genes involved in cGMP pathway (left) or spindle assembly checkpoint (SAC) machinery (center and right) in NY, SY, NO and SO granulosa cells or oocytes, respectively (n = 18 NY, 31 SY, 21 NO and 23 SO granulosa and 15 NY, 31 SY, 26 NO and 26 SO oocytes). Center line - median, box limits - first and third quartiles, whiskers - maximum and minimum or 1.5 times interquartile range if outliers, dots – outliers. **(k)** PCA of young granulosa cells computed using differentially expressed genes between NY and SY (upper panel, n = 18 NY, black, 31 SY, grey), onto which old naturally and superovulated granulosa cells are projected (lower panel, n = 21 NO and 23 SO). **(l)** PC1 coordinates of all four groups from PCA in panel k. **(m)** Quantification of shifts in PC1 between groups contrasted in panel l. For each group the mean value was used to compute the shifts. The interaction effect represents the non-additivity of aging and superovulation.

Source data are provided as a Source Data file.

Supplementary Table 1. qPCR primer design for validation of selected genes used by embryo classifier.

Target gene name	NCBI ID	Primer forward sequence	Primer reverse sequence
Kmt2a	NM_001357549.2	GGAGCGAGAGAAGGAGAATAAG	CCGACCCACAGGATACAAAG
Dync1h1	NM_030238.2	ACCTAACGGTGTTGTCTTGG	GACTCTCTGTGTGCCGTATTT
Lrp6	NM_008514.4	GAAGATGGGAGGGTAGCAATAC	GGGAAGCCCACCAGATAAAG
Ireb2	NM_022655.4	CTCACCCAGAAACAGTGAACTA	GGAGGCAGAATCACAAGATACA
Hook3	NM_207659.3	TGGCATTAGCAAGGGATGAG	CCTGGTTAGAAGGCACAGAAA
Fndc3b	NM_001356953.1	GAGATCACGGATGGAACTTCTG	GCCTGGTTTCAAATGGGTAAAG
Bptf	NM_001359590.2	CTGGGACAAGAAGGGAAGTATC	GCCTCCTCTTATCATGGTCTTC
Zfp451	NM_001359274.1	GGTGACACGTTTCTCCAACTA	GACGCACTGCTAAGGAAAGA
Nrip1	NM_173440.3	CTTTCCTATCTGGGTCAACATCTC	TGCTAAGTGGCCCTGTTATTC
Phf20l1	NM_001418886.1	TCAGAAGTCACAGGGAGTAGAA	ACTACTTTGGAGGTGGGATTTG
Vcan	NM_019389.2	AGTGGAGGAGGAAGGTATGT	GCTTCGGGATGGGTAGTATATG
Dag1	U48854.2	TCCCACTTCAGATGGTTGTG	GGAGAGATGGCTCAGTGTTAAG
GAPDH	M32599.1	GTGGCAAAGTGGAGATTGTTG	CGTTGAATTTGCCGTGAGTG

Supplementary References

1. Lee, K., Cho, K., Morey, R. & Cook-Andersen, H. An extended wave of global mRNA deadenylation sets up a switch in translation regulation across the mammalian oocyte-to-embryo transition. Cell Rep. **43**, (2024)
2. Ntostis, P. et al. The impact of maternal age on gene expression during the GV to MII transition in euploid human oocytes. Hum. Reprod. **37**, (2022).
