# Supplementary material for: Granulosa cell transcription is similarly impacted by superovulation and aging and predicts early embryonic trajectories
Source: Nat Commun. 2025 Apr 17;16:3658. doi: 10.1038/s41467-025-58451-9 (PMC12006393; doi:10.1038/s41467-025-58451-9)
Supplement: Supplementary file 3 — Description of Additional Supplementary Files [file 41467_2025_58451_MOESM3_ESM.pdf]

Description of Additional Supplementary Files

Supplementary Data 1. Quality control table for SMART-Seq2 samples (gene expression and in vitro fertilization).

Supplementary Data 2. Pairing of oocytes and granulosa cell samples used in the gene expression analyses.

Supplementary Data 3. Cell-type-specific marker genes identified from our data.

Supplementary Data 4. Overrepresentation analysis for oocytes and granulosa cells.

Supplementary Data 5. Quality control table for total RNAseq samples.

Supplementary Data 6. Overrepresentation analysis of genes differentially expressed between S and SN granulosa cells.

Supplementary Data 7. Mice numbers and ovulation rates for naturally and superovulated young and old mice.

Supplementary Data 8. Gene set enrichment analysis result table for human versus mouse granulosa cells comparison (only pathways that were significant in at least one species are included).

Supplementary Data 9. Additional information about patients undergoing IVF.

Supplementary Movie 1. Example movie for microsurgical cumulus-oocyte complex isolation, and oocyte and associated granulosa cell singularization.
