# Supplementary material for: Granulosa cell transcription is similarly impacted by superovulation and aging and predicts early embryonic trajectories
Source: Nat Commun. 2025 Apr 17;16:3658. doi: 10.1038/s41467-025-58451-9 (PMC12006393; doi:10.1038/s41467-025-58451-9)
Supplement: Supplementary file 14 — Reporting Summary [file 41467_2025_58451_MOESM14_ESM.pdf]

Corresponding author(s): Duncan T. Odom, Angela Gonçalves
Last updated by author(s): Sep 26, 2024

Reporting Summary

Nature Portfolio wishes to improve the reproducibility of the work that we publish. This form provides structure for consistency and transparency in reporting. For further information on Nature Portfolio policies, see our Editorial Policies and the Editorial Policy Checklist.

Statistics

For all statistical analyses, confirm that the following items are present in the figure legend, table legend, main text, or Methods section.

n/a	Confirmed	
☐	☒	The exact sample size (*n*) for each experimental group/condition, given as a discrete number and unit of measurement
☐	☒	A statement on whether measurements were taken from distinct samples or whether the same sample was measured repeatedly
☐	☒	The statistical test(s) used AND whether they are one- or two-sided<br>*Only common tests should be described solely by name; describe more complex techniques in the Methods section.*
☐	☒	A description of all covariates tested
☐	☒	A description of any assumptions or corrections, such as tests of normality and adjustment for multiple comparisons
☐	☒	A full description of the statistical parameters including central tendency (e.g. means) or other basic estimates (e.g. regression coefficient) AND variation (e.g. standard deviation) or associated estimates of uncertainty (e.g. confidence intervals)
☐	☒	For null hypothesis testing, the test statistic (e.g. *F*, *t*, *r*) with confidence intervals, effect sizes, degrees of freedom and *P* value noted<br>*Give P values as exact values whenever suitable.*
☒	☐	For Bayesian analysis, information on the choice of priors and Markov chain Monte Carlo settings
☐	☒	For hierarchical and complex designs, identification of the appropriate level for tests and full reporting of outcomes
☐	☒	Estimates of effect sizes (e.g. Cohen's *d*, Pearson's *r*), indicating how they were calculated

Our web collection on statistics for biologists contains articles on many of the points above.

Software and code

Policy information about availability of computer code

Data collection	Imaging data for HCR RNA FISH was acquired using Zen Black (v. 2.3, Zeiss) software, captured micrographs of embryos were processed using Zen Blue software (v.3.3., Zeiss) and cropped using Microsoft Photos App. The qPCR primers were designed using IDT PrimerQuest Tool, available online [https://eu.idtdna.com/PrimerQuest/Home/Index]. The qPCR measurements were obtained using Design & Analysis software 1 and 2 (Thermo Fisher).
Data analysis	Code used in this study is available in github repository: https://github.com/goncalves-lab/Daugelaite-Lacour-Winkler-et-al.<br>For data analysis following software, tools and packages were used (in alphabetical order, by category):<br>ImageJ (v.1.53);<br>R (v.4.0.0 and v.4.3.0);<br>SCENIC (v.1.2.4);<br>Seurat (v.4.0.3);<br>STAR (v.2.7.0f and v.2.5.3a);<br>Cogent NGS Analysis Pipeline (CogentAP, Takara Bio, v.1.5.1);<br>DKFZ ODCF RNAseqWorkflow pipeline (v.1.3.0);<br>MsigDB database [https://www.gsea-msigdb.org/gsea/msigdb/];<br>AUCell package (v.1.10.0);<br>Caret package (v.6.0-94);<br>DESeq2 (v.1.28.1);<br>EntropyExplorer package (v.1.1);<br>fgsea package (v.1.14.0);<br>glmmTMB package (v.1.1.7);

InferCNV package (v.1.17.0);
lme4 package (v.1.1.27.1);
SCnorm package (v.1.10.0);
Slingshot package (v.1.6.1).

For manuscripts utilizing custom algorithms or software that are central to the research but not yet described in published literature, software must be made available to editors and reviewers. We strongly encourage code deposition in a community repository (e.g. GitHub). See the Nature Portfolio guidelines for submitting code & software for further information.

Data

Policy information about availability of data

All manuscripts must include a data availability statement. This statement should provide the following information, where applicable:

- Accession codes, unique identifiers, or web links for publicly available datasets
- A description of any restrictions on data availability
- For clinical datasets or third party data, please ensure that the statement adheres to our policy

The primary data generated in this study have been deposited in the ArrayExpress database under accession numbers: paired oocytes and granulosa cells SMART-seq2: E-MTAB-13479 [https://www.ebi.ac.uk/biostudies/arrayexpress/studies/E-MTAB-13479]; paired embryos and granulosa cells SMART-seq2: E-MTAB-13480 [https://www.ebi.ac.uk/biostudies/arrayexpress/studies/E-MTAB-13480]; total RNA-seq in oocytes: E-MTAB-13474 [https://www.ebi.ac.uk/biostudies/arrayexpress/studies/E-MTAB-13474]; processed human granulosa cell RNA-seq: E-MTAB-13496 [https://www.ebi.ac.uk/biostudies/arrayexpress/studies/E-MTAB-13496].

Pseudonymized patient background clinical information are available under restricted access due to data protection of the participants. The access in compliance with data protection, and for research purposes only, may be obtained by contacting Dr. Julia Rehnitz (Julia.Rehnitz@med.uni-heidelberg.de) and will be processed within 3 months. Data will be available for ten years once access has been granted. Data that allows conclusions to be drawn about individuals will not be shared. This data will not be shared to third parties. The raw human RNA sequencing data are available under restricted access through the EGA database under accession number EGAD50000001210 [https://ega-archive.org/datasets/EGAD50000001210]. Access may be granted for research purposes only and in compliance with data protection regulations after signing a collaboration contract with Dr. Julia Rehnitz. The use of this data will be limited to research on oocyte and granulosa biology, and no genotyping will be allowed. Data access requests have to be submitted through the EGA portal.

The processed datasets are publicly available from Lee et al. Supplementary Data S1 [https://ars.els-cdn.com/content/image/1-s2.0-S221112472400038X-mmc2.xlsx], Xue et al. from ArrayExpress with accession number E-GEOD-44183 [https://www.ebi.ac.uk/biostudies/arrayexpress/studies/E-GEOD-44183], and Ntostis et al. from NCBI Gene Expression Omnibus repository with accession number GSE164371 [https://www.ncbi.nlm.nih.gov/geo/query/acc.cgi?acc=GSE164371].

The additional data generated in this study are provided in the Supplementary Information/Source Data files. Sequencing quality controls, pairing of oocytes, embryos with their granulosa cells, cell type specific marker genes, overrepresentation analyses for filtered genes, gene set enrichment analyses, information about mice and basic patient background information are available in Supplementary Data 1-9. Source data is available for all relevant figures presented in this study.

Research involving human participants, their data, or biological material

Policy information about studies with human participants or human data. See also policy information about sex, gender (identity/presentation), and sexual orientation and race, ethnicity and racism.

Reporting on sex and gender	The sex of all participants was female, as assigned by the presence of reproductive organs (ovaries, uterus, vagina). No information on gender identity of the participants was obtained.
Reporting on race, ethnicity, or other socially relevant groupings	No information on ancestry or socioeconomic status of the participants was collected or used in this research.
Population characteristics	Age of the participants ranges from 27 to 45. All participants were normal responders to in vitro fertilization (IVF) and were referred for IVF due to male or idiopathic subfertility.
Recruitment	Patients undergoing their first cycle of in vitro fertilization (at the University Women's Hospital Heidelberg, Heidelberg, Germany, were selected for this study. Only normal responder patients that were referred for IVF due to male or idiopathic subfertility were included in this study.
Ethics oversight	All patient procedures were approved by the local ethical committee of the University of Heidelberg, Germany (Ethikkommission der Medizinische Fakultät Heidelberg, study approval number S-602/2013). Informed written consent about clinical procedures, pseudonymized data usage and completed clinical questionnaires were signed by all participating patients. Participants did not receive any compensations.

Note that full information on the approval of the study protocol must also be provided in the manuscript.

Field-specific reporting

Please select the one below that is the best fit for your research. If you are not sure, read the appropriate sections before making your selection.

☒ Life sciences ☐ Behavioural & social sciences ☐ Ecological, evolutionary & environmental sciences

For a reference copy of the document with all sections, see nature.com/documents/nr-reporting-summary-flat.pdf

Life sciences study design

All studies must disclose on these points even when the disclosure is negative.

Sample size	No statistical method was used to predetermine sample size, however, a minimum of 3 individuals per experimental group was used for statistical testing and reproducibility, similar to previous studies (Pan et al., 2008; Lee et al., 2017).<br>References:<br>Pan, H., Ma, P., Zhu, W. & Schultz, R. M. Age-associated increase in aneuploidy and changes in gene expression in mouse eggs. Dev. Biol. (2008). doi:10.1016/j.ydbio.2008.01.048.<br>Lee, M. et al. Adverse effect of superovulation treatment on maturation, function and ultrastructural integrity of murine oocytes. Mol. Cells (2017). doi:10.14348/molcells.2017.0058.
Data exclusions	The data exclusion criteria were based on fixed quality control metrics evaluated after sequencing. Mice oocyte, embryo and granulosa cell samples were discarded: if the number of reads was below 5000, if the number of genes detected was below 1000, or if the percentage of mitochondrial reads was above 5% (Supplementary Data 1). Ten samples were re-sequenced due to metadata mis-assignment. Human granulosa cell samples were filtered based on mitochondrial reads percentage (below 18%, corresponding to 80% percentile) to remove low quality samples (4 samples in total). To disregard outliers in the HCR RNA FISH staining quantification, cells that were more than two standard deviations above the mean fluorescence intensity were excluded.
Replication	Each experiment could be reproduced on a different experimental day, sometimes separated over a few years ,for both mice and human patients.
Randomization	The experiments were not specifically randomized. Mice were ordered from an external provider on different dates. Each experimental group contains mice from different orders. This was not tracked during analysis as the strain is highly inbred. Human patients could not be randomized due to the nature of treatments performed.
Blinding	The investigators were not blinded to the group allocation during data collection or analysis. Blinding during data collection was not possible due to the protocol. Analysis was performed on all groups simultaneously using the same scripts so blinding was either irrelevant or not possible.

Reporting for specific materials, systems and methods

We require information from authors about some types of materials, experimental systems and methods used in many studies. Here, indicate whether each material, system or method listed is relevant to your study. If you are not sure if a list item applies to your research, read the appropriate section before selecting a response.

Materials & experimental systems

n/a	Involved in the study	
☒	☐	Antibodies
☒	☐	Eukaryotic cell lines
☒	☐	Palaeontology and archaeology
☐	☒	Animals and other organisms
☒	☐	Clinical data
☒	☐	Dual use research of concern
☒	☐	Plants

Methods

n/a	Involved in the study	
☒	☐	ChIP-seq
☒	☐	Flow cytometry
☒	☐	MRI-based neuroimaging

Animals and other research organisms

Policy information about studies involving animals; ARRIVE guidelines recommended for reporting animal research, and Sex and Gender in Research

Laboratory animals	Mice were kept at the DKFZ animal facility in Tecniplast GM500 IVC cages in groups of up to six mice under controlled light-dark cycle (12h/12h, from 7:00 to 19:00), at ambient temperature of 20-23oC and 60-70% humidity. The animals had access to standard laboratory chow, water and environmental enrichments ad libitum. None of the animals were involved in previous procedures. Mus musculus mice, specifically, CD-1 male and C57BL/6J female mice were purchased from Janvier, France, and allowed to adapt to animal facility conditions for at least 1 week, C57BL/6Ly5.1 female mice were bred in house at DKFZ animal facility. All female mice used in experiments for cell collection were euthanized by cervical dislocation, CD-1 male mice were euthanized by carbon dioxide when they were no longer suitable for sham-mating. Natural or superovulation was performed on young (11-14 weeks) and old (50-58 weeks) female mice, where young mice were all C57BL/6J and old mice were all C57BL/6Ly5.1.
Wild animals	No wild animal was used in this study.
Reporting on sex	This study was on oocytes and granulosa cells and was thus only performed on female mice. Sex was determined based on anogenital distance at birth and the presence of female reproductive organs during experiments.
Field-collected samples	No field-collected samples were used in this study.

Ethics oversight	All animal experiments were carried out according to governmental and institutional guidelines and approved internally by the animal welfare officer (Tierschutzbeauftragter, approval number DKFZ-366) and by the local authorities (Regierungspräsidium Karlsruhe, approval number G-238/19).

Note that full information on the approval of the study protocol must also be provided in the manuscript.

Plants

Seed stocks	*Report on the source of all seed stocks or other plant material used. If applicable, state the seed stock centre and catalogue number. If plant specimens were collected from the field, describe the collection location, date and sampling procedures.*
Novel plant genotypes	*Describe the methods by which all novel plant genotypes were produced. This includes those generated by transgenic approaches, gene editing, chemical/radiation-based mutagenesis and hybridization. For transgenic lines, describe the transformation method, the number of independent lines analyzed and the generation upon which experiments were performed. For gene-edited lines, describe the editor used, the endogenous sequence targeted for editing, the targeting guide RNA sequence (if applicable) and how the editor was applied.*
Authentication	*Describe any authentication procedures for each seed stock used or novel genotype generated. Describe any experiments used to assess the effect of a mutation and, where applicable, how potential secondary effects (e.g. second site T-DNA insertions, mosiacism, off-target gene editing) were examined.*
